# Detection of *Mycobacterium leprae* DNA from Archaeological Skeletal Remains in Japan Using Whole Genome Amplification and Polymerase Chain Reaction

**DOI:** 10.1371/journal.pone.0012422

**Published:** 2010-08-26

**Authors:** Koichi Suzuki, Wataru Takigawa, Kazunari Tanigawa, Kazuaki Nakamura, Yuko Ishido, Akira Kawashima, Huhehasi Wu, Takeshi Akama, Mariko Sue, Aya Yoshihara, Shuichi Mori, Norihisa Ishii

**Affiliations:** 1 Laboratory of Molecular Diagnostics, Department of Mycobacteriology, Leprosy Research Center, National Institute of Infectious Diseases, Tokyo, Japan; 2 School of Rehabilitation Sciences at Fukuoka, International University of Health and Welfare, Fukuoka, Japan; 3 Department of Pharmacology, National Research Institute for Child Health and Development, Tokyo, Japan; 4 Laboratory of Molecular Epidemiology and Social Science, Leprosy Research Center, National Institute of Infectious Diseases, Tokyo, Japan; 5 Leprosy Research Center, National Institute of Infectious Diseases, Tokyo, Japan; New Mexico State University, United States of America

## Abstract

**Background:**

Identification of pathogen DNA from archaeological human remains is a powerful tool in demonstrating that the infectious disease existed in the past. However, it is very difficult to detect trace amounts of DNA remnants attached to the human skeleton, especially from those buried in a humid atmosphere with a relatively high environmental temperature such as in Asia.

**Methodology/Principal Findings:**

Here we demonstrate *Mycobacterium leprae* DNA from archaeological skeletal remains in Japan by polymerase chain reaction, DNA sequencing and single nucleotide polymorphism (SNP) analysis. In addition, we have established a highly sensitive method of detecting DNA using a combination of whole genome amplification and polymerase chain reaction, or WGA-PCR, which provides superior sensitivity and specificity in detecting DNA from trace amounts of skeletal materials.

**Conclusion/Significance:**

We have detected *M. leprae* DNA in archaeological skeletal remains for the first time in the Far East. Its SNP genotype corresponded to type 1; the first detected case worldwide of ancient *M. leprae* DNA. We also developed a highly sensitive method to detect ancient DNA by utilizing whole genome amplification.

## Introduction

Leprosy is a chronic infectious disease caused by *Mycobacterium leprae* (*M. leprae*) and has affected humans for millennia. Whole genome sequence analysis of *M. leprae* has revealed that the genome is 3.3 Mbp in size, with only 1,605 genes that encode proteins and 1,115 pseudogenes [1]. Trough comparative genome analysis of *M. leprae* strains from Brazil, India, Thailand and United States, remarkably little genomic diversity has been uncovered suggesting that leprosy has a single clonal origin [2], [3]. Phylogeographic analysis of single nucleotide polymorphisms (SNPs) has revealed that *M. leprae* originated in Africa and spread to European and Asian countries and then worldwide along with human migrations and trade routes. Such geographic and temporal migration has also been demonstrated by SNPs analysis of ancient *M. leprae* DNA present in skeletal remains as old as 1,500 years ago [2], [3].

Leprosy results in multiple deformities including progressive bone defects secondary to the peripheral neuropathy caused by *M. leprae* infection [4], [5]. Additionally, in advanced cases *M. leprae* infection causes specific osteological deformations in the areas of the nasal aperture, anterior nasal spine and alveolar process on the premaxilla, cortical areas of the tibia and fibula, distal ends of the metatarsals and diaphyses of the phalanges that may include both direct and reactive changes [6], [7], [8], [9], [10]. Paleopathological diagnosis of leprosy has been made solely based on these macroscopic changes in skeletal remnants. However, it is occasionally difficult to make a definitive paleopathological diagnosis of leprosy solely based on osseous lesions, because syphilis, tuberculosis and other infectious and granulomatous diseases as well as carcinomas of nasal or oral origin will cause rhinomaxillary lesions similar to leprosy.

Direct detection of DNA remnants of pathogenic microorganisms from ancient human skeletons using polymerase chain reaction (PCR) is becoming a powerful molecular tool for clearly demonstrating the presence of pathogens within the skeletal remains [11]. This method provides an inestimable tool for investigating the temporal and geographical spread of infectious diseases in human history, in addition to the anthropological interest in diagnosing infectious diseases in ancient remains. By the use of so-called palaeomicrobiology, it may be possible to elucidate the epidemiology of past infectious disease by reconstituting the distribution of infected individuals, which is especially important in the field of emergence and re-emergence of infectious diseases [11]. Moreover, it may also be possible to track the genetic evolution of the pathogenic microorganisms.

A study employing paleomicrobiology was first reported in 1993, demonstrating the presence of *Mycobacterium tuberculosis* (*M. tuberculosis*) DNA using PCR from an ancient human skeleton [12]. Detection of *M. leprae* DNA was reported the next year [13] from ancient bone dating from 600 AD, followed closely by several other reports [14], [15], [16], [17], [18], [19], [20], [21]. However, all of these studies used European and Middle Eastern archaeological materials from countries such as England, Germany, Denmark, Poland, Hungary, Czech, Croatia, Turkey, Israel and Egypt, but not from the Far East Asian countries. This may be related to the superior preservation of ancient buried skeletons in these countries compared to that in more a humid atmosphere with a relatively high environmental temperature, such as is commonly found in Asia [22].

Recently, we have obtained permission to examine skeletal remains excavated in Japan with paleopathological evidence of leprosy In the present study, we demonstrate *M. leprae* DNA and SNPs from skeletal remains. We also describe a highly sensitive whole genome amplification (WGA)-PCR method that may be suitable for detecting trace amounts of ancient microbial DNA.

## Materials and Methods

### Archaeological background and osteological description

**Figure 1:**
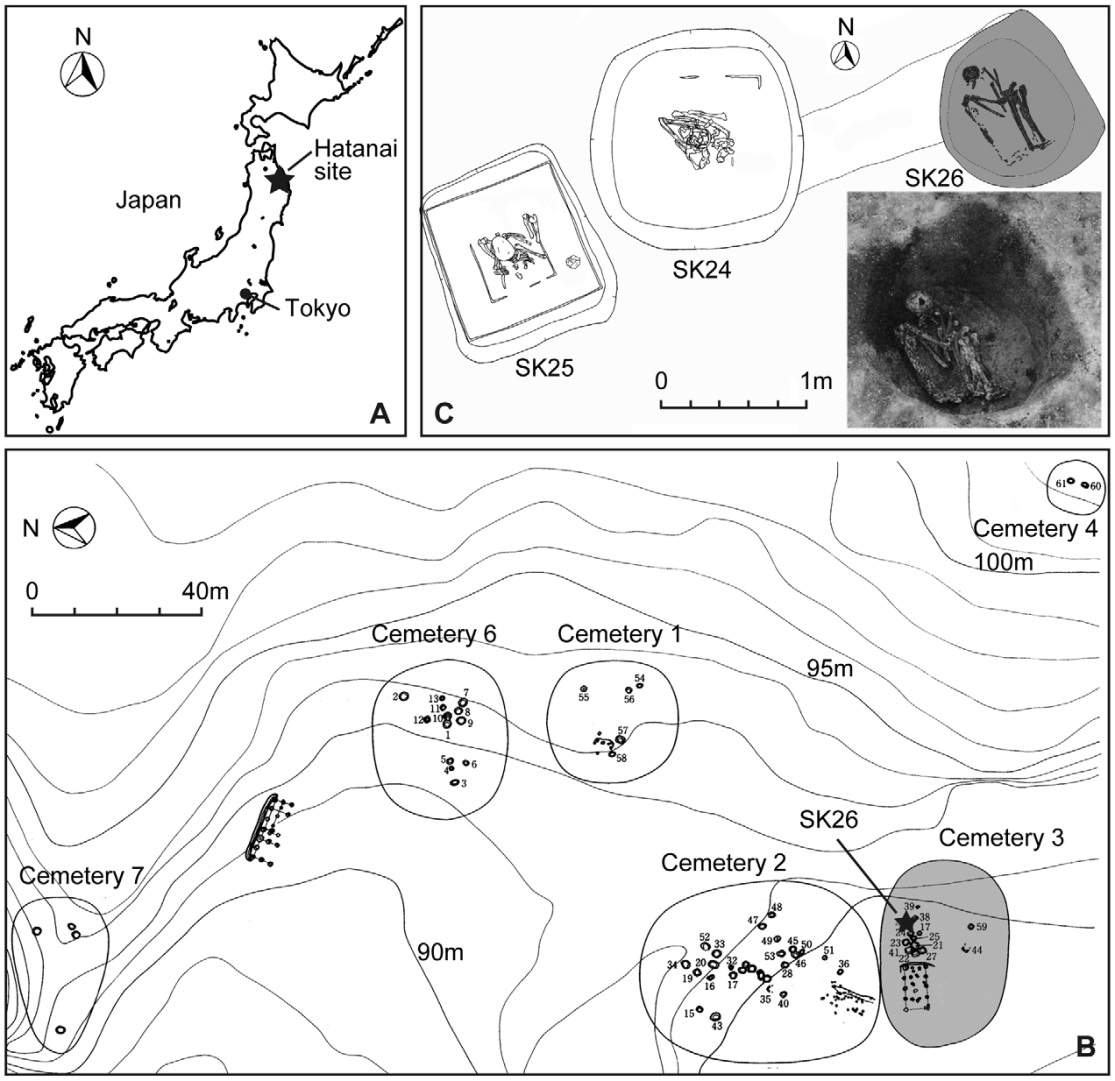
Geographical representation of excavation site. Location of *Hatanai* site in northeastern Japan (A). A map of seven cemeteries and the site where SK26 was excavated (B). A grave pit of SK26 at excavated (C) Cemetery 5 (not shown in this map) locates 50 m south of cemetery 2. All these figures were modified with permission from reference [23].

The materials used in this study were archaeological skeletal remains excavated from the *Hatanai* site (N40°22′, E141°29′) in Aomori prefecture, in the northeastern part of Honshu Island in Japan (Fig. 1A). The *Hatanai* site consists of several strata including artifacts and remnants from the prehistoric Initial-Middle Jomon (ca. 8000-4500 BP) to pre-modern Edo period (1603–1867). All skeletal materials are stored in the Tohoku University Museum, Sendai, Japan.

The present material (Grave No. SK26) was buried at a gravel pit of an Edo-period cemetery in a farm village, in which was found about 50 human skeletal remains (Fig. 1B). This corpse was lying on its left side and hunching its back, with the elbow, hip and knee joints closely flexed inside the burial pit (Fig. 1C). It is not clear whether or not SK26 was placed in a wooden coffin. The SK26 skeleton had only one *Kouki-tsuhou (Kangxi-tongbao)*, a metallic currency used during the rule of the Emperor Kangxi (reign in 1661–1722) of the Qing dynasty (1616–1912) in China, as grave goods. Accordingly, the age of this burial is estimated as the middle 18th to the early 19th century, as supposed by evidences from other graves [23].

**Figure 2:**
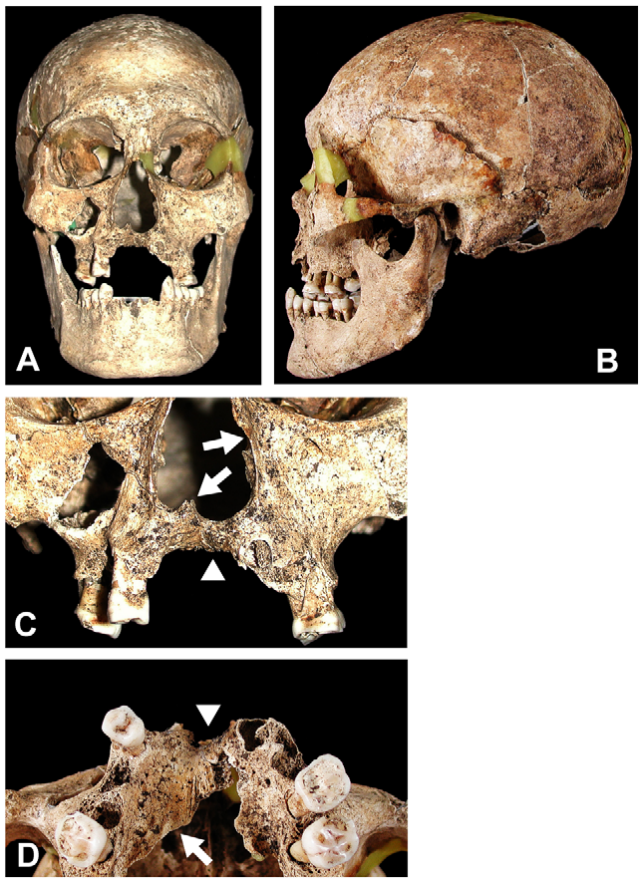
Macroscopic view of the skeletal lesion in the skull of sample SK26. Frontal view (A) and left side view (B) of the skull. Closer view of the nasal aperture and maxillary palate (C) and upward view of the palate (D). Arrows in panel C indicate erosive deformity of the nasal aperture and disappearance of the anterior nasal spine. Arrowheads in panels C and D indicate severe atrophy of alveolar bone in the maxilla/palatal process with loss of anterior teeth. An arrow in panel D denotes the locus of sample No. 1 shown in Table 1 and Figure 2.

SK26's skull was almost perfectly intact except for part of the cranial base and parietal bones (Fig. 2). Although its main long bones were intact, many of their epiphyses and all the hand/foot bones were missing within the burial matrix of the postmortem environment. The sex of this skeleton was assessed as male on the basis of cranial morphologies, such as anterior decline of the frontal bone, projection of the superciliary ridge, inferior prominence of the mastoids, and developments of the superior nuchal line and the external occipital protuberance. But its bilateral pelvis was incompletely preserved and therefore unavailable for sex and age determination.

The age at death was estimated from dental attrition and fusions of the cranial sutures. Surviving permanent teeth were worn to dentine, including the 3rd molar. In the calvarium, the unions of all sutures in the internal table were finished; coronal and lambdoid sutures of the external table had incomplete fusing. These dental and skeletal evidences suggest that of the age of death, SK26 was middle-aged, in the range of 30–50 years old.

### Palaeopathological observations and diagnosis

The facial cranium of SK26 showed several typical symptoms of leprosy, suggestive of lepromatous type. In the rhinomaxillary area, rounding deformation and abnormal enlargement of the nasal aperture, disappearance of the anterior nasal spine, and severe atrophy of the alveolar process were clearly observed (Fig. 2). The palatal process showed degenerative changes, and alveolar bones at the anterior teeth were closed by antemortem loss of all upper incisors and the right canine. The incisor foramen in the hard palate had also disappeared due to osseous resorption; for maxillary atrophy, the vertical thickness of the palatal bone was about nine millimeters on the right side of the alveolar process, and at the same portion on the left, only six millimeters at the maximum value.

These features of craniofacial malformation, especially the rhinomaxillary syndrome, are specific to lepromatous leprosy but essentially distinct from other facial-deforming diseases such as craniofacial tuberculosis and syphilis, a treponematoses [7], [8]. Although the nasal, maxillary and palatal lesions in craniofacial tuberculosis closely resemble those in lepromatous leprosy, cranial involvements of tuberculosis are extremely rare in adult patients and mainly appear in young children, often with lesions of the mandible. In tertiary syphilis, rhinomaxillary deformations accompany several osteo-periostitic lesions and focal destructive remodeling (caries sicca) caused by the bone gummas in the cranial vault [7], [8]. From differences in these pathological features, the facial deformities of SK26 were diagnosed as leprous symptoms.

In the bilateral tibiae and fibulae of this skeleton, slight periostitic changes were seen on the cortical surfaces of the diaphyses. The SK26 skeleton had lost its hand and foot bones from the long-term burial conditions, making it impossible to confirm any pencil-shaped recessions in the distal ends of the metacarpals, metatarsals or phalanges, which are typical leprous lesions.

### Sampling of skeletal remains

Sampling was performed at the Tohoku University Museum, where no other leprous materials were stored. Skeletal samples were taken from the affected area as summarized in Table 1 using a small-sized rotating electric saw (Mini router, Kiso Power Tool, Osaka, Japan). Two samples were also taken from another skeleton (SK20; middle-aged male) found in the same cemetery, which had no leprous changes as a negative control for DNA purification and PCR analysis. One premolar root was extracted from another skeleton (SK16; middle-aged female) as a positive control for human DNA recovery. Sterile materials were used for the sampling to avoid possible contamination. Ethical approval to work with the material was obtained from the review board at National Institute of Infectious Diseases, Japan. A permission to obtain the sample materials was granted by Tohoku University.

**Table 1:** Skeletal samples.

No.	Material reference	Sampling site	Paleopathological evidence	Sample weight (mg)
1	SK26	Maxillary palate, right	Erosion/atrophy	9.7
2	SK26	Inner surface of nasal cavity, right	Erosion/atrophy	6.0
3	SK26	Maxillary palate, left	Erosion/atrophy	4.6
4	SK26	Fibular diaphysis, right	Periostitis	22.1
5	SK20	Maxillary palate, right	N.R.	4.5
6	SK20	Inner surface of nasal cavity, right	N.R.	6.8
7	SK16	Lower 1st premolar root, right	N.R.	3.4

N.R.: No remarkable change.

### DNA preparations

DNA was purified using a QIAamp DNA Micro Kit (Qiagen, Valencia, CA) according to the manufacturer's protocol with some modification. Briefly, 500 µl of Buffer ATL was added to the skeletal samples placed in a 2 ml test tube. The tube was then filled with 1.0 mm diameter Zicronia beads (Bio Space Products, Inc, South Lancaster, MA) and homogenized using Micro Smash MS-100 (TOMY, Tokyo, Japan) at 3,000 rpm for 5 min at 4°C [24], [25]. Samples were then frozen at −80°C, thawed at 37°C and homogenized for five times. The supernatant was subsequently treated with 40 µl of Proteinase K (20 mg/ml) and incubated overnight at 56°C. The samples were frozen and thawed, vortexed in 400 µl of Buffer AL then 400 µl of ethanol was added and incubated for 5 min. Then the mixture was applied to a QIAamp MinElute column and centrifuged at 6,000× g for 1 min at room temperature. Flow-through was discarded and the column was subsequently washed and DNA was eluted by adding 20 µl of Buffer AE and centrifugation at 20,000× g for 1 min.

### Polymerase chain reaction (PCR) and DNA sequencing

Touchdown PCR was performed using a PCR Thermal Cycler DICE (TaKaRa, Kyoto, Japan) as described previously [26]. PCR primers were designed to be specific to the *M. leprae* genome and were used in previous studies [24], [27], [28]; all the primers are listed in Table 2. The PCR products were analyzed by 2% agarose gel electrophoresis. Nested PCR was used to amplify *hsp-70*, *16S rRNA* and SNPs. Some of the results were confirmed in an independent laboratory.

**Table 2:** PCR primer sequences.

Gene name	Synonym^a)^	Forward (5′-3′)	Reverse (5′-3′)	Length (bp)
Nested primer				
*hsp-70*, 1st	ML2496	gggctgtccaaggaagag	cgtcaaccacatcgtcagtag	391
*(dnaK)* 2nd		tcgtcaaggaacaacggg	cgtcaaccacatcgtcagtaga	261
*16S rRNA*, 1st	MLP000016	agagtttgatcctggctcag	tgcacacaggccacaaggga	1039
(*rrs*) 2nd		cggaaaggtctctaaaaaatctt	catcctgcaccgcaaaaagctt	171
SNP1, 1st	14,676	atttccaggtcttgtgcg	aggtcttcccaggacacc	351
2nd		aatggaatgctggtgagagc	caatgcatgctagccttaatga	194
SNP2, 1st	1,642,875	ttagtcaacatcgttagcagccc	actcataagcacggtgtctttgc	403
2nd		tgctagtttaaccgagtactgcta	gtagtagtcttccaagttgtggtg	189
SNP3, 1st	2,935,685	cgagcataatcgtaggcg	aaatgtggtcacctgggc	510
2nd		atctggtccgggtaggaatc	accggtgagcgcactaag	180
Gene				
*cspA*	ML0198	gaactgtgaagtggttcaacg	agcgaactccagtggcttg	186
*hisE*	ML1309	gaccttcgaggatctgttcg	atagacgtcatcgagcgaca	247
*purM*	ML2205	aaactcacgtccgtaccttc	agcacggtcagtatcttcag	233
*cpsA*	ML2247	ggcaccttcaccaacctaga	ccaacttaggatccgcttga	511
Pseudogene				
*pseudo scoB*	ML0434	tggaacacctcgtcgtatgtgg	tataagtggcaccgccgaactc	201
*pseudo speE*	ML0476	tcgcaacttcactgatcgtc	gtctggcaccaataccgagt	465
*pseudo REP*	ML0794	aaagacggagactacgatg	gtttagaaggttggtcgttg	191
*pseudo ahpD*	ML2043	tcaacatggcgatctgcattc	tgcgtgaccttacaacgct	200
Non-coding region				
	348,457	tggactcgatgttgaagtg	tgcttagctatgcagtgag	202
	1,593,211	catcgagtccaagctcaac	tgccgatgattacatcatcc	191
	2,134,972	cggaatcctgttgacgtgtt	cggcgctaacaactatcctc	242
	2,152,288	ccgatatgttcggtagtcgt	gcatcgatatcgccttcag	241
	2,307,322	ggttcaccggaagagttgg	cgcgacgactaagccagtag	242
	2,546,884	tcaatatggcttcctatgttgc	gctgcattaatccatgattcg	202
	2,551,060	acattcgagaccagctaccg	ttccgcttggaggataattg	194
	2,664,658	tgagcttgccgattacgatt	gccattgaactggccatc	227
	2,858,681	atgttggttgagcttggac	ttgcttagctatgcagtgag	217

Numbers in SNP nested primer indicate the coordinate location of the SNP. Numbers in non-coding regions indicate coordinate position of the primer within *M. leprae* genome (http://genolist.pasteur.fr/Leproma/).

The DNA sequence was analyzed using an ABI PRISM 310 Genetic Analyzer and GeneScan Collection software (Applied Biosystems).

### Whole genome amplification (WGA)

A GenomePlex Whole Genome Amplification kit (Sigma, St Louis, MO) was used to amplify genomic DNA samples according to the manufacturer's protocol [29]. Briefly, 1 µl of 10× Fragmentation Buffer was mixed with 10 µl of DNA solution and incubated for 4 min at 95°C. After cool down, 2 µl of 1× Library Preparation Buffer and 1 µl of Library Stabilization Solution were added and incubated for 2 min at 95°C. Then 1 µl of 1× Library Preparation Enzyme was added and incubated at 16°C for 20 min, at 24°C for 20 min, at 37°C for 20 min and at 75°C for 5 min to ensure library preparation. To amplify genomic DNA, 7.5 µl of 10× Amplification Master Mix, 47.5 µl of Nuclease-free Water and 5 µl of Jumpstart *Taq* DNA polymerase were added and heated at 95°C for 3 min, then 14 cycles of two-temperature amplification was employed at 94°C for 15 sec and 65°C for 5 min using a PCR Thermal Cycler DICE (TaKaRa). The quality of the WGA products was evaluated by 1% agarose gel electrophoresis.

### Others

All experiments were performed in a biosafety level 2 (BSL2) laboratory where samples were handled in a safety cabinet using disposable sterile materials to avoid any contamination.

## Results

### Demonstration of *M. leprae* DNA by PCR and DNA sequencing

**Figure 3:**
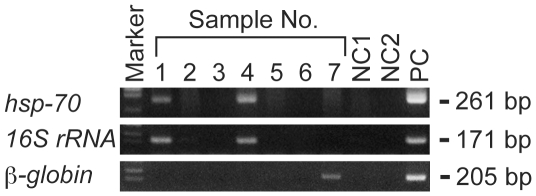
PCR detection of *M. leprae* DNA from skeletal samples. PCR analysis was performed using *M. leprae*-specific *hsp-70* and *16S-rRNA* primers for the DNA samples listed in Table 1. PCR products were evaluated by 2% agarose gel electrophoresis. Human *β-globin* gene was also PCR amplified as a control for DNA preparation from a premolar root. NC1: a negative control for DNA purification in which DNase/RNase-free water was used as a sample for DNA extraction; NC2: a negative control for PCR in which DNase/RNase-free water was used instead of a DNA sample for PCR reaction; and PC: positive control DNA from *Thai 53* strain of *M. leprae*.

The purified DNA from seven different sites of skeletons from three individuals was subjected to PCR amplification of the *M. leprae*-specific *hsp-70* gene and *16S rRNA* genes. A nested PCR protocol to amplify the *M. leprae*-specific *16S rRNA* region was developed in our laboratory. Among four samples taken from leprous lesions, a specific positive signal was derived from sample No. 1 (right maxillary palate of SK26) and No. 4 (right fibula of SK26), but not from the others (Fig. 3). Samples taken from control bones were all negative. Human *β-globin* DNA was detected by PCR only from sample No. 7 (lower premolar root of SK16).

The specificity of these PCR amplifications was confirmed by DNA sequencing of the PCR products purified from agarose gel. Basic Local Alignment Search Tool (BLAST) search of the DNA sequence was a 100% match with the reported *M. leprae* sequence for both *hsp-70* and *16S rRNA* as expected (data not shown). The results clearly confirmed that *M. leprae* was involved in the skeletal lesions of SK26 diagnosed as lepromatous leprosy.

### SNP analysis of the *M. leprae* DNA

**Figure 4:**
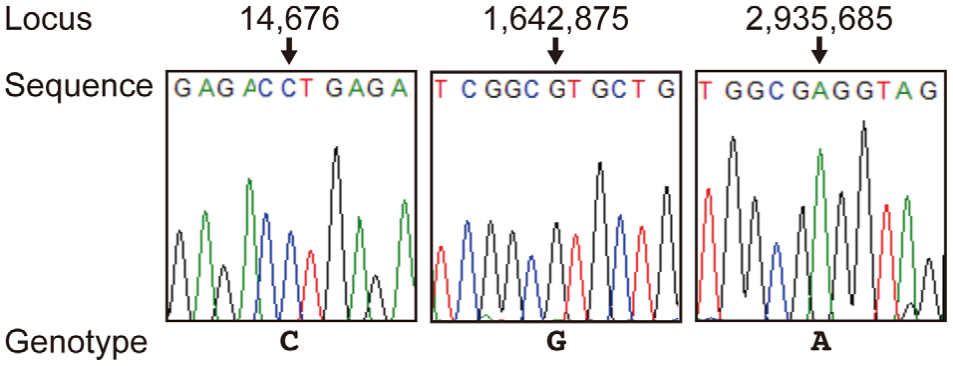
Sequence analysis of the three reported SNPs of *M. leprae* DNA. Each locus was PCR amplified and sequenced as described in the Materials and Methods.

To determine the possible origin of the *M. leprae* found in this skeleton, we analyzed single-nucleotide polymorphisms (SNPs) at 3 reported loci in the *M. leprae* genome [2], [3]. PCR amplification followed by direct sequencing identified the sequence at the 3 loci. Positions 14,676, 1,642,875 and 2,935,685, were “C”, “G” and “A”, respectively (Fig. 4). This genotype of *M. leprae* DNA is reported as SNP type 1, which is the dominant type in India and Southeast Asia [2], [3].

### Evaluation of WGA-PCR method

**Figure 5:**
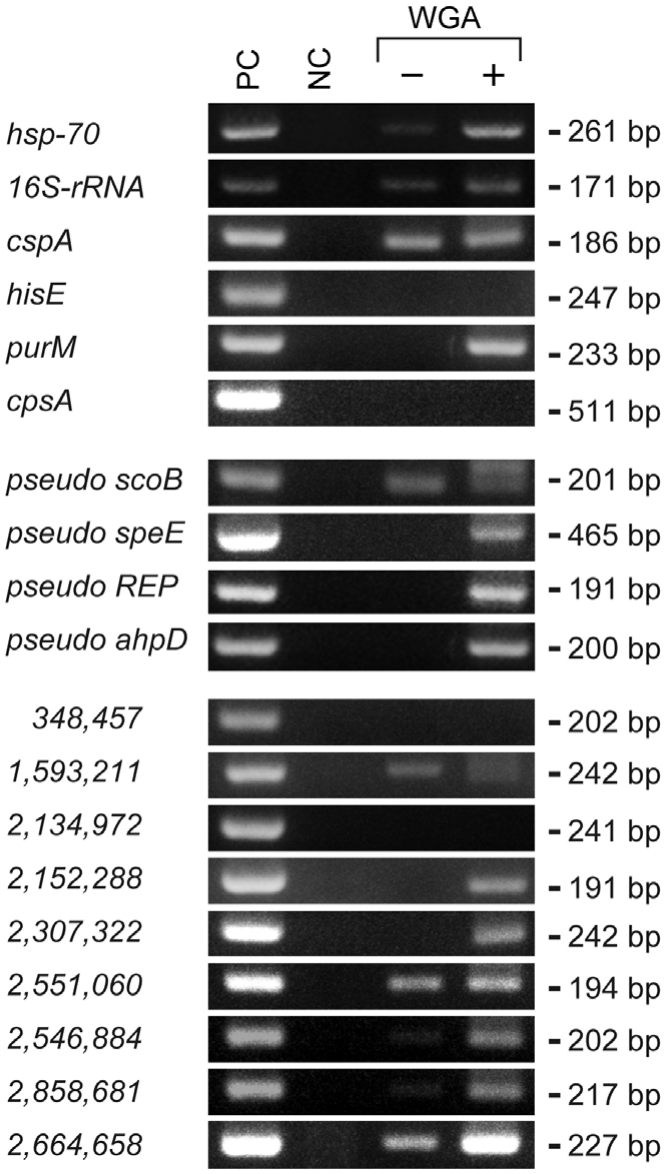
Comparison of conventional PCR and WGA-PCR. PCR was performed using a DNA sample derived from the present study (sample No. 1 shown in Table 1 and Fig. 3). The DNA before and after WGA (“−” and “+”, respectively) was amplified using specific nested primers and direct primers targeted for the genes, pseudogenes and non-coding regions of *M. leprae* genome as listed in Table 2. Names of the genes and pseudogenes are indicated and the coordinate positions of non-coding regions in the genome (http://genolist.pasteur.fr/Leproma/) are indicated numerically. PC: positive control DNA from *Thai 53* strain of *M. leprae*; and NC: negative control using DNase/RNase-free water instead of a DNA sample for PCR reaction.

We have attempted to amplify other regions of the *M. leprae* genome using PCR primers we have designed previously [27] and found that the sensitivity to detect *M. leprae* DNA from ancient samples is significantly lower than from the clinical samples we analyze in our laboratory. To solve this problem, we have applied WGA to amplify whole genomic DNA purified from skeletal samples. Purified DNA from sample No. 1 was subjected to WGA and the DNA before and after WGA was compared by PCR using 19 different PCR primers targeting open reading frames, pseudogenes and non-coding regions of the *M. leprae* genome [24], [27]. WGA-amplified DNA was positive for 15 sets of PCR primers, while *M. leprae* genomic DNA was detected by only 7 primer sets in the original DNA sample (Fig. 5), showing that WGA-PCR has superior sensitivity over conventional PCR.

## Discussion

We have demonstrated *M. leprae* DNA in excavated human skeletal remnants from Japan by both PCR analysis and DNA sequencing. We also sequenced three SNPs in the *M. leprae* DNA and confirmed that the bacilli are of the dominant type in Southeast Asia. Although *M. leprae* DNA has already been found in archaeological bone remains in Europe and the Middle East [13], [14], [15], [16], [17], [18], [19], [20], [21], there have been no reports from Far East Asia. This may be related to the geographical and environmental diversity between Europe and Asia, where archaeologists and anthropologists have not paid much attention to palaeopathological topics of leprosy and other infectious diseases [22]; Asia's predominantly hot and humid climate may cause difficulty in recovering skeletons buried for several hundred years.

The present case was excavated in the northern part of Japan and showed typical osteological changes of leprosy. We took skeletal samples from four different areas, the inner surface of the nasal cavity, maxillary palate and diaphysis of the fibula, that showed deformities by erosion and atrophy, or mild periostitis. *M. leprae* DNA was successfully demonstrated in the maxillary palate and fibular diaphysis.

Although subperiostal exostoses and/or hypertrophy accompanied by swelling or porotic hyperosteosis of the fibula are reported as the typical signs of leprosy [6], [7], the presence of *M. leprae* DNA in the fibula was somewhat surprising to us because apparent fibular lesion is not common in leprosy patients at present. It is well known that the common fibular nerve is one of the sites preferably affected by *M. leprae,* which causes foot drop in the patient [30]. It may therefore be possible to speculate that *M. leprae* affected the periosteum through this nerve. If that is the case, the present identification of *M. leprae* DNA in the fibula supports the possibility that *M. leprae* can spread significantly throughout the body when patients are left untreated.

The SNP genotype of *M. leprae* DNA in *Hatanai* SK26 was type 1, which is a major group in Southeast Asia and India, but rather minor in modern East Asia including Japan and China [2], [3]. All reported SNP types of ancient *M. leprae* DNA from Europe and Middle East are type 3, which is also a major genotype in the same regions at present [3]. However, this study confirmed the first case of SNP type 1 as ancient DNA samples. Although more evidence is required, this suggests the possibility that in the Far East inclusive of Japan, the dispersal process of the phylogenetic type of leprosy is historically different from the occidental world. In the Southeast Asia and India where the modern dominant SNP group is type 1, archaeological skeletal remains with lesions of lepromatous leprosy have been excavated and dated from ca. 2000 BC (India) to ca. 300 BC-500 AD (Thailand) [31], [32]; therefore the type 1 genotype in modern Japan might be traced back to Southeast Asia and probably originated in India. A historical record of ancient Japan mentioned that the oldest case suggestive of leprosy dated back to the 7^th^ century, and the oldest positive palaeopathological evidence at present is dated to the 13^th^ century. The genotypes of these ancient and medieval materials are still unknown, and further examination of ancient materials from Eastern and Southeast Asia are needed in order to determine the dispersal history of leprosy in Far East Asia.

In our laboratory, PCR detection of *M. leprae* DNA is routinely performed from small amounts of clinical samples of skin smears or paraffin sections as part of a government reference center. It is difficult, however, to apply the same PCR protocol to detect *M. leprae* from ancient DNA recovered from skeletal remains. To overcome this problem, we have utilized WGA, a technique that enables amplification of minute amounts of DNA [29], to increase the sensitivity for PCR detection. We have successfully utilized this method to amplify and detect environmental bacteria from small amounts of water taken from the sea or lakes in Japan (unpublished data).

By PCR reaction using WGA-amplified DNA (WGA-PCR method), large amounts of DNA could be generated from minute amounts of original DNA samples and the detection rate of *M. leprae* DNA and signal intensity were significantly improved. Thus, this method enables highly sensitive detection of small amounts of clinical, environmental and ancient DNA. On the other hand, it also carries the risk of contamination and false positives, particularly if performed by an individual lacking appropriate knowledge and training in molecular biology. Even using this method, however, some primers still did not work on ancient DNA. This might be due to DNA fragmentation in the ancient sample and/or because of the DNA fragmentation process employed during the library preparation step in the WGA protocol. In general, genomic DNA from dead organisms gradually degrades into short fragments over time mainly due to the activity of nucleases produced by microorganisms in the soil. PCR detection, even using the present WGA-PCR method, will be difficult to perform when such a fragmentation proceeds, especially for very old materials and those buried in conditions where the decomposition process is accelerated, *e.g.* a hot and humid environment. Nevertheless, WGA-PCR seems to be advantageous for improving the sensitivity of DNA recovery from these samples. We are now routinely utilizing this method to detect ancient DNA and believe that it will be applicable for detecting other kinds of DNA from archaeological samples, as well as other kinds of samples with trace amounts of DNA.
